# Modifying the severity and appearance of psoriasis using deep learning to simulate anticipated improvements during treatment

**DOI:** 10.1038/s41598-025-91238-y

**Published:** 2025-03-03

**Authors:** Joseph Scott, James A. Grant-Jacob, Matthew Praeger, George Coltart, Jonathan Sutton, Michalis N. Zervas, Mahesan Niranjan, Robert W. Eason, Eugene Healy, Ben Mills

**Affiliations:** 1 Dermatology, University Hospital Southampton NHS Foundation Trust, Southampton, SO16 6YD UK; 2 Dermatopharmacology, Faculty of Medicine, University of Southampton, Southampton, SO16 6YD UK; 3 Optoelectronics Research Centre, University of Southampton, Southampton, SO17 1BJ UK; 4 Learning and Control Group, University of Southampton, Southampton, SO17 1BJ UK

**Keywords:** Psoriasis, Personalised medicine, Generative artificial intelligence, Image processing, Deep learning, Neural network

## Abstract

A neural network was trained to generate synthetic images of severe and moderate psoriatic plaques, after being trained on 375 photographs of patients with psoriasis taken in a clinical setting. A latent w-space vector was identified that allowed the degree of severity of the psoriasis in the generated images to be modified. A second latent w-space vector was identified that allowed the size of the psoriasis plaque to be modified and this was used to show the potential to alleviate bias in the training data. With appropriate training data, such an approach could see a future application in a clinical setting where a patient is able to observe a prediction for the appearance of their skin and associated skin condition under a range of treatments and after different time periods, hence allowing an informed and data-driven decision on optimal treatment to be determined.

**Supplementary Information:**

The online version contains supplementary material available at 10.1038/s41598-025-91238-y.

## Introduction

Psoriasis is an inflammatory skin disorder that affects 2.8% of people in the U.K. (i.e., > 1 million individuals) and is estimated to affect between 65 to 125 million people globally (noting that 81% of countries worldwide lack information on psoriasis epidemiology)^1–3^. It frequently presents as erythematous, scaly plaques on the skin that are different in colour to the surrounding healthy skin. Various treatments are available for psoriasis including topical agents (creams and ointments), ultraviolet radiation (phototherapy) and systemic agents (traditional systemics such as methotrexate, ciclosporin, acitretin, and biologic and biosimilar medications such as anti-tumour necrosis factor, anti-interleukin-12/23, and anti-interleukin-17 agents)^4^. Choosing the most appropriate therapy is a careful balance between the severity of the disease, the benefits of using a particular treatment modality and the risks of adverse effects of that treatment. Currently, such decisions are made in tandem by the medical practitioner and the patient, and therefore subject to bias and incomplete information. There is therefore a clear need for a data-driven approach that allows patients in a clinical environment to visualise personalised, AI digital twin images of the appearance of their skin under different treatment plans. This would allow an informed and balanced decision regarding the optimal treatment (while also bearing in mind the risk of potential adverse effects of the different treatments) and hopefully also increase adherence to the optimal treatment.

Deep learning has become a critically important tool for a wide range of image processing applications, enabling the capability for solving extremely challenging tasks without the need for a programmatical description of the desired process, and hence can provide scientific insight in situations with complex datasets. Recent applications of deep learning include microscopy^5^, sensing^6^, and within the field of medicine^7^. As the treatment approach for psoriasis, and indeed other skin conditions, is determined based on a visual inspection of the patient’s skin by a medical practitioner, it is understandable that deep learning has already been applied to the identification of psoriasis in photographs. Recent examples include automated labelling of psoriasis boundary and severity^8–10^, and the automated labelling of psoriasis for the potential of targeted irradiation^11^.

Deep learning can also be used to generate synthetic images. In this work, we combine this ability with the concept of ‘latent w-space’ to deliberately modify features in generated images of psoriasis, to demonstrate the potential for simulating the appearance of a patient’s skin under different treatment conditions. This is achieved through the use of a generative neural network known as StyleGAN^12,13^ for producing a set of realistic images of psoriasis and the use of human labelling to help identify a latent w-space vector that corresponds to the change in appearance of the psoriasis during treatment. This latent w-space vector can be understood as a vector that can be added to any synthetic image, to transform a specific feature in the image. In the case of images of generated human faces, examples of transformations include changing the hair colour, facial features, pose and adding or removing accessories such as glasses^14,15^.

The work presented here represents a proof-of-concept demonstration for using a neural network to alter the appearance of psoriasis, in AI-generated images of psoriasis, similar to what we see during treatment in clinical practice. A key novel demonstration here is the method of identification of a latent w-space vector that corresponds to the reduction in severity of psoriasis in the generated images, and the application of this vector for producing realistic synthetic images that demonstrate a gradual reduction in the severity of psoriasis. The latent w-space vector was developed with the input of three dermatologists with significant experience of treating psoriasis clinically with a wide variety of therapies, including topical agents (creams and ointments), ultraviolet radiation, and systemic agents given orally, intravenously, and subcutaneously. This approach also has the potential for application to other skin diseases such as acne, eczema, alopecia, vitiligo, and any other skin condition that may benefit from a data-driven predictive visualisation tool for identification of the optimal treatment.

## Methodology

The authors acquired 375 photographs from 21 patients with psoriasis attending the Dermatology department, University Hospital Southampton NHS Foundation Trust, U.K. to create the training dataset for the neural network used in this work. All subjects participated voluntarily and provided written informed consent to participate in this study. Ethical approval for this work was obtained from the West of Scotland Research Ethics Committee (22/WS/0015 299722) and all experiments were performed in accordance with the relevant regulatory approvals.

Photographs were taken using an Apple iPad, with an emphasis on the collection of photographs from different angles, distances, and lighting levels. Whilst the filenames included an anonymised patient ID number, this information was not made available to the neural network. The photographs were augmented to 25,000 images via random cropping, rotation, inversion, perspective warp, and changing the RGB values to simulate different lighting levels, and finally resized to 256 × 256 pixels. This variation in lighting as well as how the image is visualised is relevant to the clinical situation. This is because the lighting in clinic rooms and treatment rooms varies according to the use of combinations of artificial and natural light sources over the course of each day and during the seasons. Moreover, individual psoriatic lesions in patients with multiple plaques of psoriasis would be visualised by the viewer/camera at different angles/perspectives, thus it is important that the model is able to deal with these aspects in the clinic.

In this study we used the StyleGAN2-ADA network which was selected to specifically address the challenges of discriminator overfitting in limited data scenarios, which was a concern given the limited availability of clinical data for this proof-of-principal demonstration. The full details of the implementation of this network, and code, is provided by Karras et al.^16^. In this network, the generator produces synthetic data from latent variables, which is passed through an augmentation module before being evaluated by the discriminator. Real data undergoes the same augmentation process, ensuring consistent treatment of both real and generated samples. The discriminator is trained with losses that aim to distinguish between real and augmented generated data, while the generator updates its parameters to maximize the discriminator’s error. This augmentation mechanism is adaptive *(ADA (Adaptive Discriminator Augmentation))* and stabilises training without modifying the loss functions or network architectures and is particularly effective in scenarios where data is scarce. The hyperparameters used for this study are as follows: 1. Learning Rate: Generator: lr = 0.0025, Discriminator: lr = 0.0025. 2. Batch Size: batch_size = 64. 3. Latent Dimensions: z_dim = 512, w_dim = 512. 4. Resolution: resolution = 256. 5. Channel Base and Max: Generator: channel_base = 16,384, channel_max = 512; Discriminator: channel_base = 16,384, channel_max = 512. 6. Loss Function Parameter: r1_gamma = 0.2048. 7. Total Training Duration: total_kimg = 5000.

## Results and discussion

**Fig. 1:**
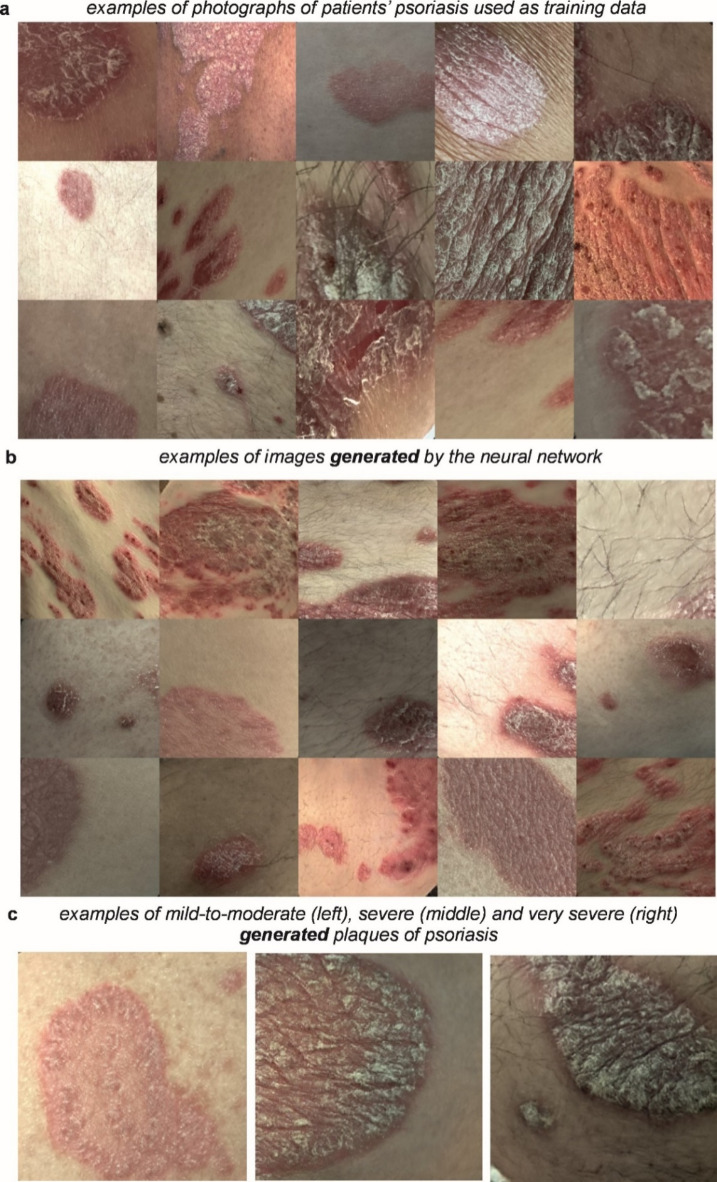
Showing (**a**) photographs of patients’ psoriasis taken in a clinical setting, (**b**) images of psoriasis generated by the neural network and (**c**) examples of generated plaques of psoriasis of different disease severity.

The neural network was trained on the 25,000 augmented images, taking 28 h, using dual Intel® Xeon® Gold 5222 CPUs at 3.80 GHz and three NVIDIA RTX 4500 GPUs. Once trained, the neural network was used to generate synthetic images of psoriasis. Figure 1 shows 15 examples of (a) patient photographs and (b) images generated by the neural network. The images in the figure were randomly chosen and convey the wide distribution of image data used for, and generated from, this approach. The figure shows a clear similarity between patient images and generated images, and indeed other features such as human hair are also present in some of the generated images. We did not use any filtering to detect and exclude artifacts, such as blurring, and no post-processing was applied to generated images. Additional examples of real patient photographs and images of generated psoriasis are shown in Supplementary Fig. 1 and Supplementary Fig. 2. As can be seen in these examples and in Fig. 1c, the network was able to generate psoriatic lesions of different disease severity. Supplementary Fig. 3 illustrates examples of psoriatic lesions in light and dark skin, as well as generated images of psoriasis in light and dark skin, demonstrating that our model can synthesise images of psoriasis in different skin tones. We did not set out to recruit patients according to their skin tone, and further work will be required to ensure that there is sufficient diversity in our dataset so that the model is able to recognise and generate images of psoriasis in each of the different skin tone groups.

### Analysis of generated images

**Fig. 2:**
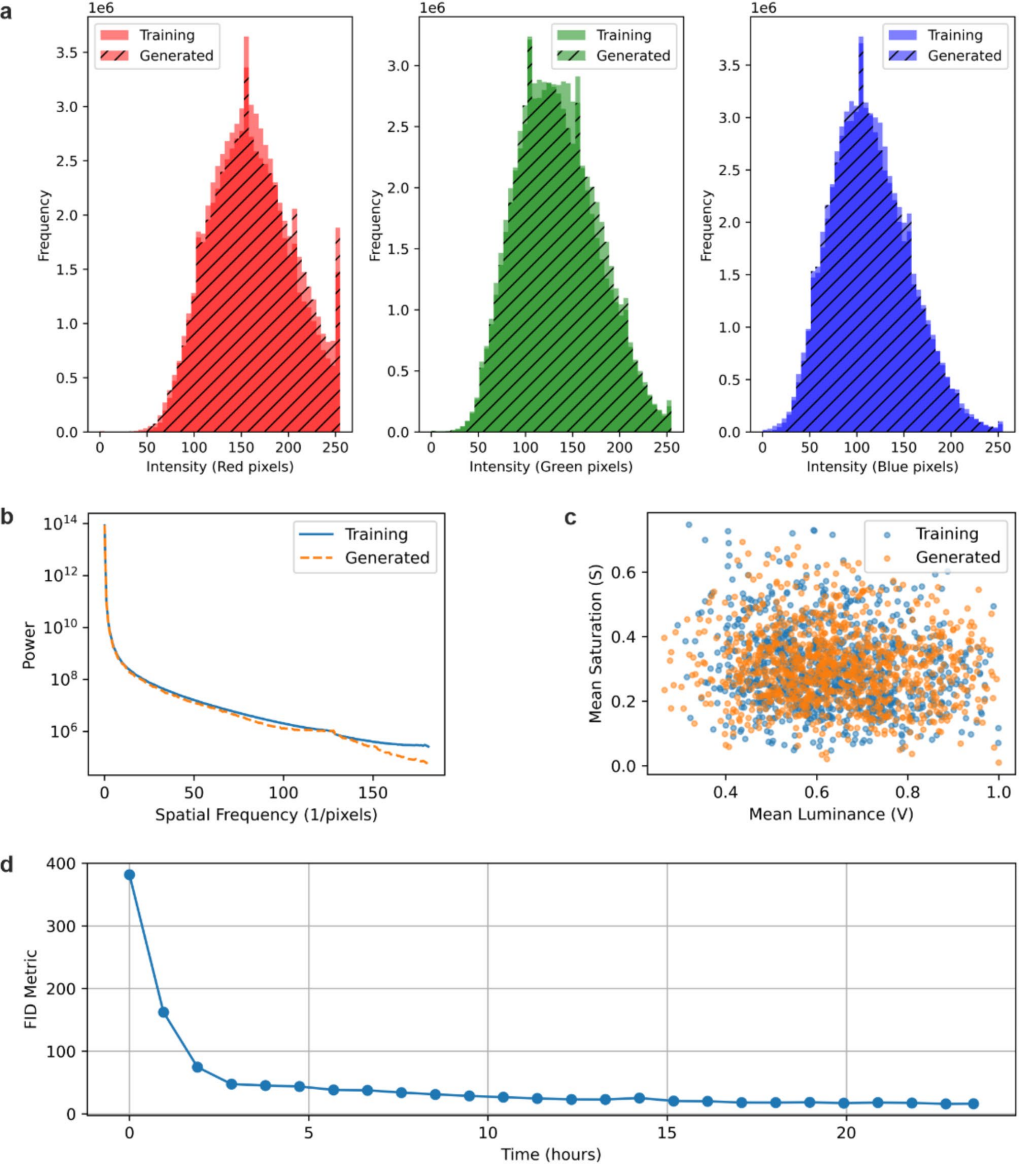
Showing (**a**) raw pixel intensities in the red, green and blue channels of training and generated images of psoriasis, (**b**) spatial frequency content in training and generated images, (**c**) dot plots of mean saturation and mean luminance, and (**d**) Fréchet Inception Distance (FID) over time during training of the StyleGAN model.

To evaluate the realism of the generated images from a statistical perspective, a range of image characterisation techniques were applied to compare a set of 1000 randomly chosen training data images with 1000 generated images. Figure 2a shows the raw pixel intensities in the red, green, and blue channels of the two sets of images. The histograms confirm that the distributions of the sets of images are broadly similar, indicating that the generative model effectively captures the fundamental colour properties and pixel-level statistics, and hence accurately reproducing the low-level colour characteristics of the underlying dataset. Figure 2b demonstrates the spatial frequency content in the images by computing the Fourier transform and averaging the power at various frequencies, hence showing how image textures and patterns are represented. The nearly overlapping curves suggest that the generative model reproduces higher-order spatial statistics and textures with considerable fidelity. Figure 2c plots mean saturation against mean luminance to compare the images in a feature space derived from colour attributes. The scattered points for both sets occupy a similar region, showing a comparable range of image colourfulness and brightness. This overlap provides further evidence that the generative model produces a broad array of colour and lighting conditions that are similar to those in the training data. Figure 2d shows the progression of the Fréchet Inception Distance (FID) metric over time during training, highlighting the effectiveness of the adaptive augmentation mechanism. The FID metric decreases sharply in the initial hours indicating rapid improvements in the quality of the generated images and then plateaus as the training stabilises and convergence is achieved. This demonstrates that the proposed training strategy can effectively reduce overfitting and produce high-quality results despite limited clinical data.

### Dermatologists’ assessment of generated images

**Fig. 3:**
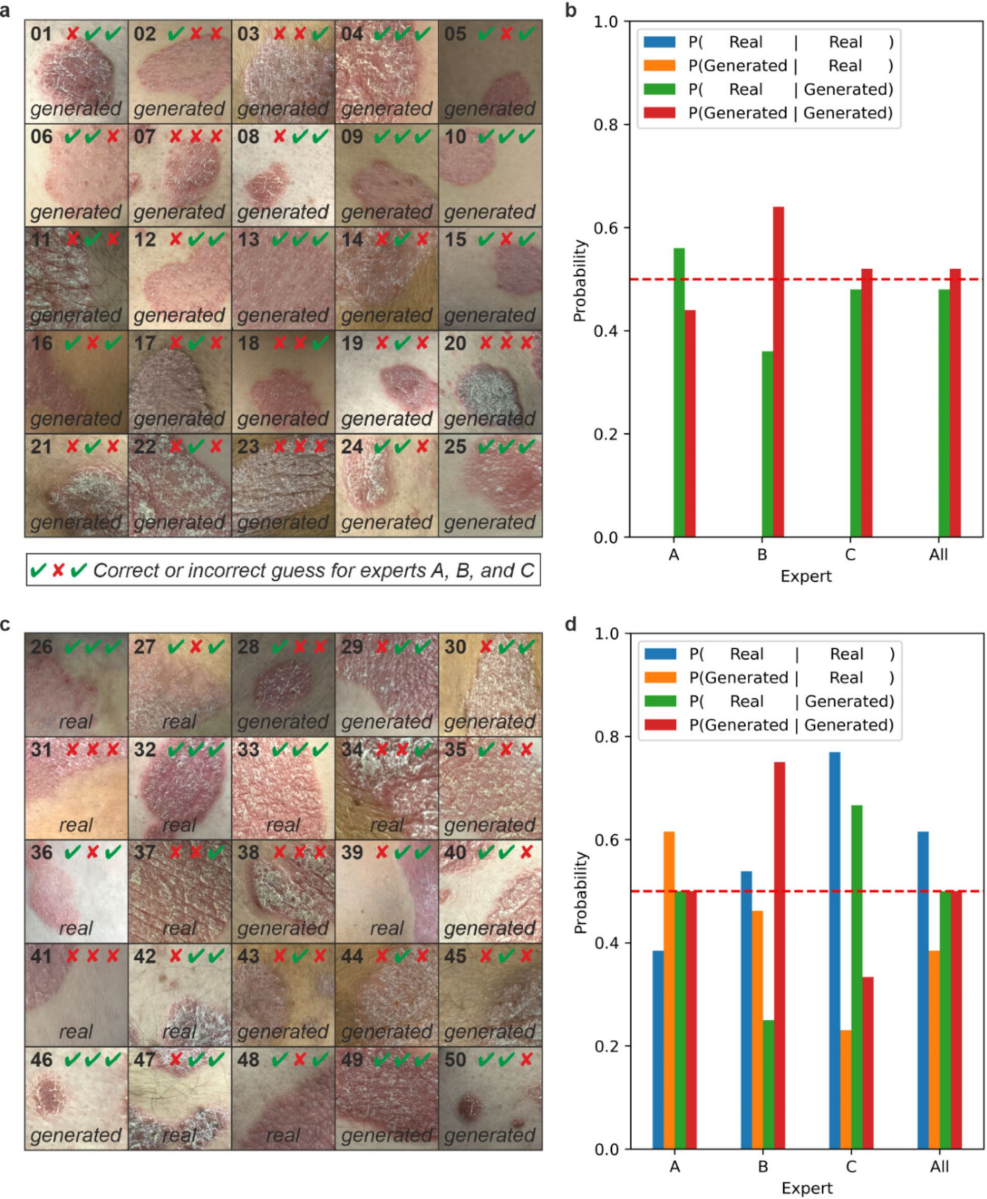
Three dermatologists (A, B and C) were asked to analyse 50 images of psoriasis and state if images were real, clinical photographs of patients’ psoriasis, or images generated by the StyleGAN network (**a**) and (**c**). Bar charts illustrating the performance of three dermatologists in (**b**) correctly identifying StyleGAN-generated images as generated images and (**d**) distinguishing real images of psoriasis from StyleGAN-generated images.

 To increase confidence in the generative model’s ability to synthesise realistic images of psoriasis, we asked three dermatologists to independently assess 50 images of psoriasis and state whether they believe the images to be real (i.e. photographs of patients’ psoriasis) or generated by the StyleGAN network. Images were all 256 × 256 in size and had not been seen previously by individuals performing the assessment. Dermatologists were not told how many of the images were real or generated within the dataset and were given sufficient time (up to one minute) to assess the images and decide. Figure 3a,c shows all 50 images used for this blinded assessment. Correct and incorrect responses given by the dermatologists are indicated by a tick or cross, respectively. The first 25 images (Fig. 3a) in this blinded assessment were images generated by the StyleGAN model, and Fig. 3b illustrates the responses of the dermatologists. The green bar represents the proportion of generated images evaluated as real by dermatologists, and the red bar represents correctly identified generated images. Figure 3c comprises 25 images of which 13 are real images of psoriasis taken from patients in a clinical setting. Overall, dermatologists correctly identified real images of psoriasis with 60% accuracy, and correctly identified network-generated images of psoriasis 50% of the time (with 50% of generated images evaluated as real by dermatologists). The three dermatologists who assessed the images in this blinded assessment had 6, 8 and 33 years’ experience in dermatology respectively, with each of these regularly seeing psoriasis in their clinical practice and with the latter dermatologist having led a complex psoriasis clinic for > 20 years. This indicates that our model generates images of psoriasis that are deemed realistic by dermatologists who have significant experience in the diagnosis and treatment of psoriasis.

### Categorisation of generated images according to severity of psoriasis

**Fig. 4:**
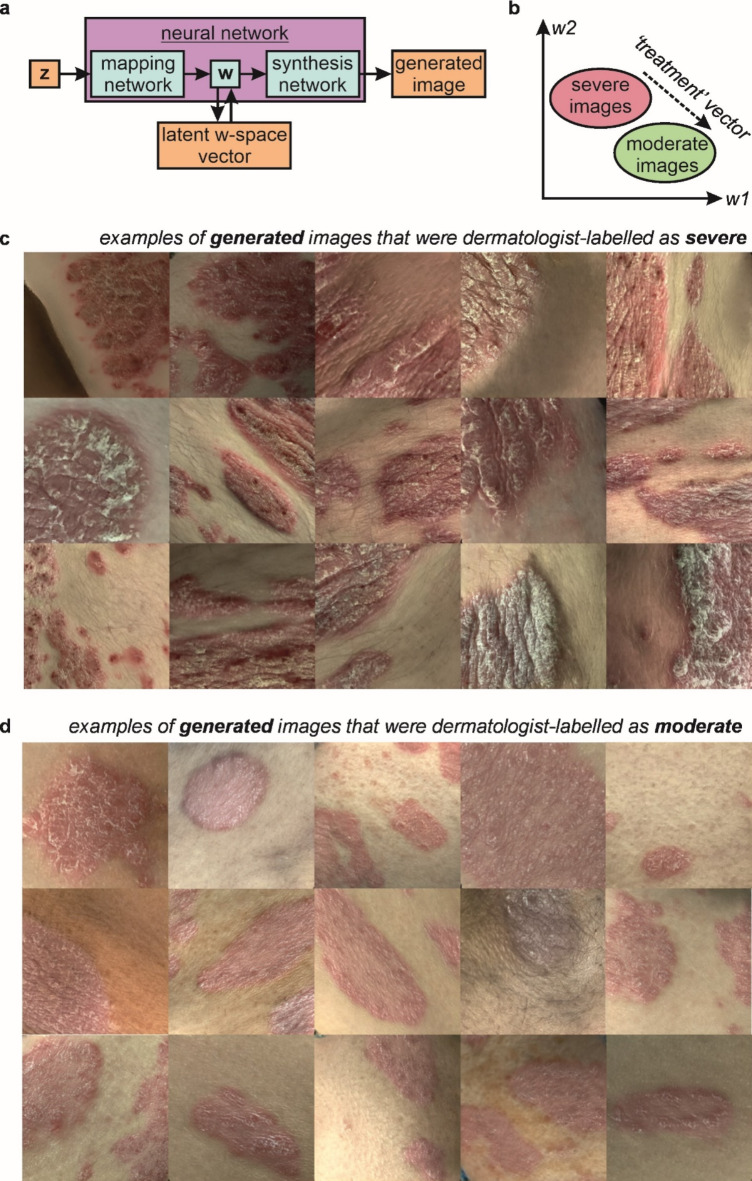
Schematics of (**a**) the neural network and (**b**) latent w-space vector identification, and 15 examples of generated images labelled by the authors as (**c**) severe and (**d**) moderate.

As shown in Fig. 4a, a key innovation in the StyleGAN is the use of two separate neural networks, referred to as the mapping network and the synthesis network, as this allows a higher-abstraction representation of the image data to be observed. The mapping network transforms the vector **z** into the vector **w**, and the synthesis network transforms the vector **w** into a generated image. Here, **z** and **w** both correspond to a vector with dimensions 1 × 512, where **z** is a latent vector in z-space and **w** in w-space, and either a **z** or a **w** vector can be used to generate an image. Critically, when a **z** vector is used to create an image, the associated **w** vector can also be observed. As shown by the concept in Fig. 4b, this means that the **w** vectors for a set of generated images labelled as severe can be compared with the **w** vectors for a set of generated images labelled as moderate, and hence allows the identification of an averaged **w** vector that maps the categories of severe onto moderate. In this work, this w-space vector is referred to as the ‘treatment’ vector, as it represents the w-space mapping that one might observe during treatment of psoriasis.

**Fig. 5:**
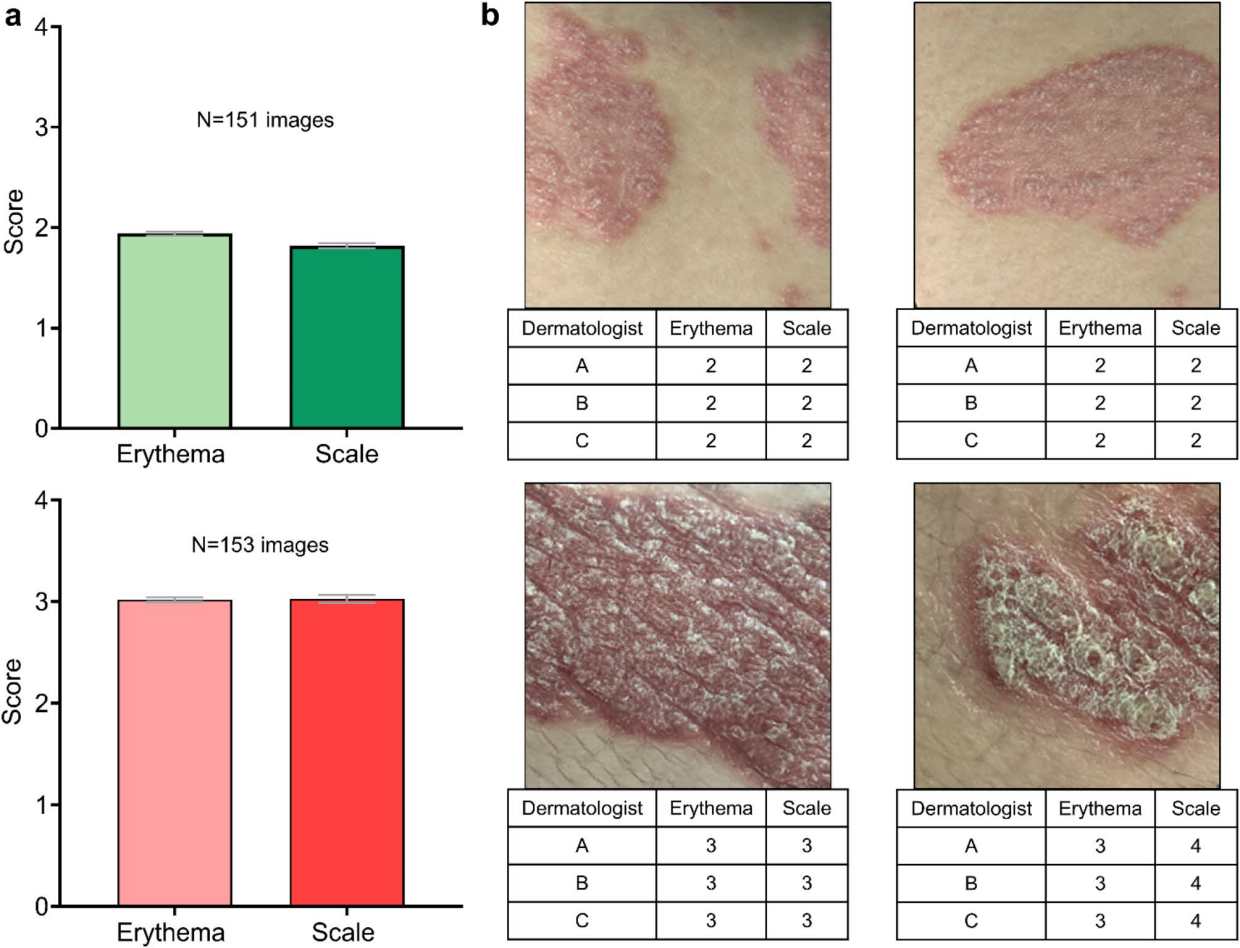
(**a**) Bar charts showing the mean erythema and scale scores, and standard error of the mean, for neural network-generated images of moderate (n = 151) and severe (n = 153) psoriasis, assessed by three dermatologists. (**b**) Examples of moderate and severe images of psoriasis generated by the neural network, with the respective erythema and scale scores as quantified by three dermatologists.

To achieve this, neural network-generated images of psoriasis were independently labelled by three dermatologists based on severity of erythema (redness) and scaling on a 0 to 4 scale (where 4 = very severe, 3 = severe, 2 = moderate, 1 = mild, 0 = none, as used for these components of the psoriasis area and severity index (PASI) which is widely employed in clinical practice) as previously described^10^. 153 images were categorised as ‘severe’, and 151 images were categorised as ‘moderate’, with 15 examples from each category shown in Fig. 4c,d. 55 images of severe psoriasis and 54 images of moderate psoriasis were used in the final analysis to generate the **w,** ‘treatment’, vector. Note that the concept in Fig. 4b is shown in 2 dimensions for simplicity, as the latent w-space had 512 dimensions. This ‘treatment’ vector could subsequently be added to any generated image of psoriasis to decrease the severity or it could be subtracted from the generated image to increase the severity. Fractions of this vector could also be applied, for example adding 50% of the vector to a generated image to achieve a 50% decrease in severity. Figure 5a shows the mean (and standard error of the mean) erythema and scale scores by three dermatologists for neural network-generated images of moderate and severe psoriasis, and Fig. 5b examples of the neural-network generated images of moderate and severe psoriasis with accompanying dermatologists’ scores.

### Demonstration of the application of the treatment vector

**Fig. 6:**
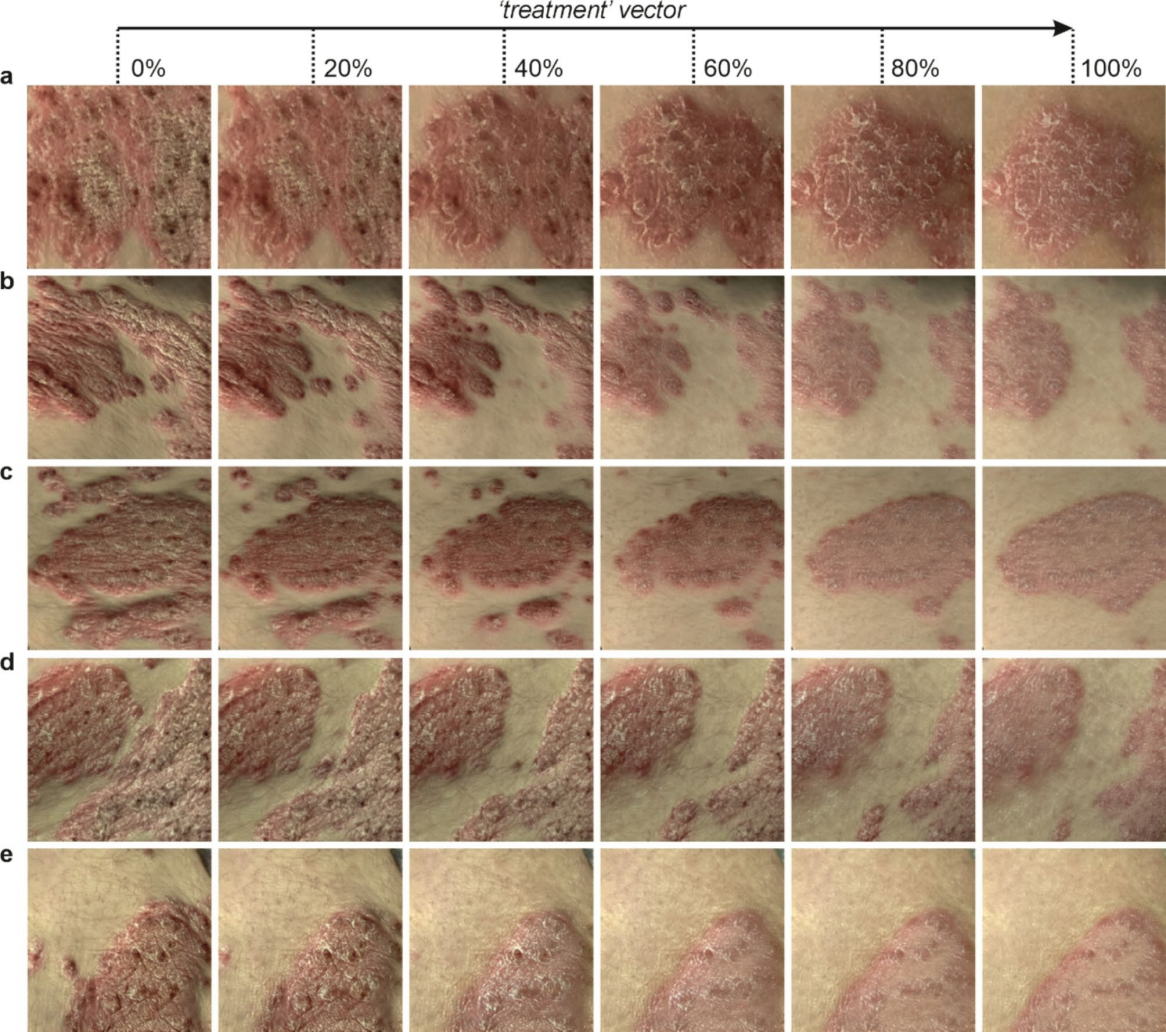
Examples of generated images with different fractions of the identified ‘treatment’ vector.

Figure 6 shows examples of adding the ‘treatment’ vector to five generated images (a–e), in fractions of 20%, 40%, 60%, 80% and 100%. Each row of images shows a clear reduction in severity, with the changes corresponding to a reduction from severe to moderate. Whilst in general the shape and size of the psoriatic region, and indeed location of hairs, remain the same during this transformation, a slight change in the size of the psoriasis plaque is observed in some cases as the ‘treatment’ vector is added. This change is attributed to a slight correlation between severity and plaque size in the training dataset, however, we know from dermatology experience that the scaling and erythema of psoriatic plaques improve during effective treatment but that the size of plaques generally remains the same during the earlier stages of improvement. Whilst training data bias, such as this observed correlation between severity of psoriasis and plaque size, could be alleviated through the collection of additional and targeted training data, the solution that is demonstrated here is to identify a second vector (in this case the ‘size’ vector) and use this to compensate for the training data bias.

### Demonstration of the application of the size vector

**Fig. 7:**
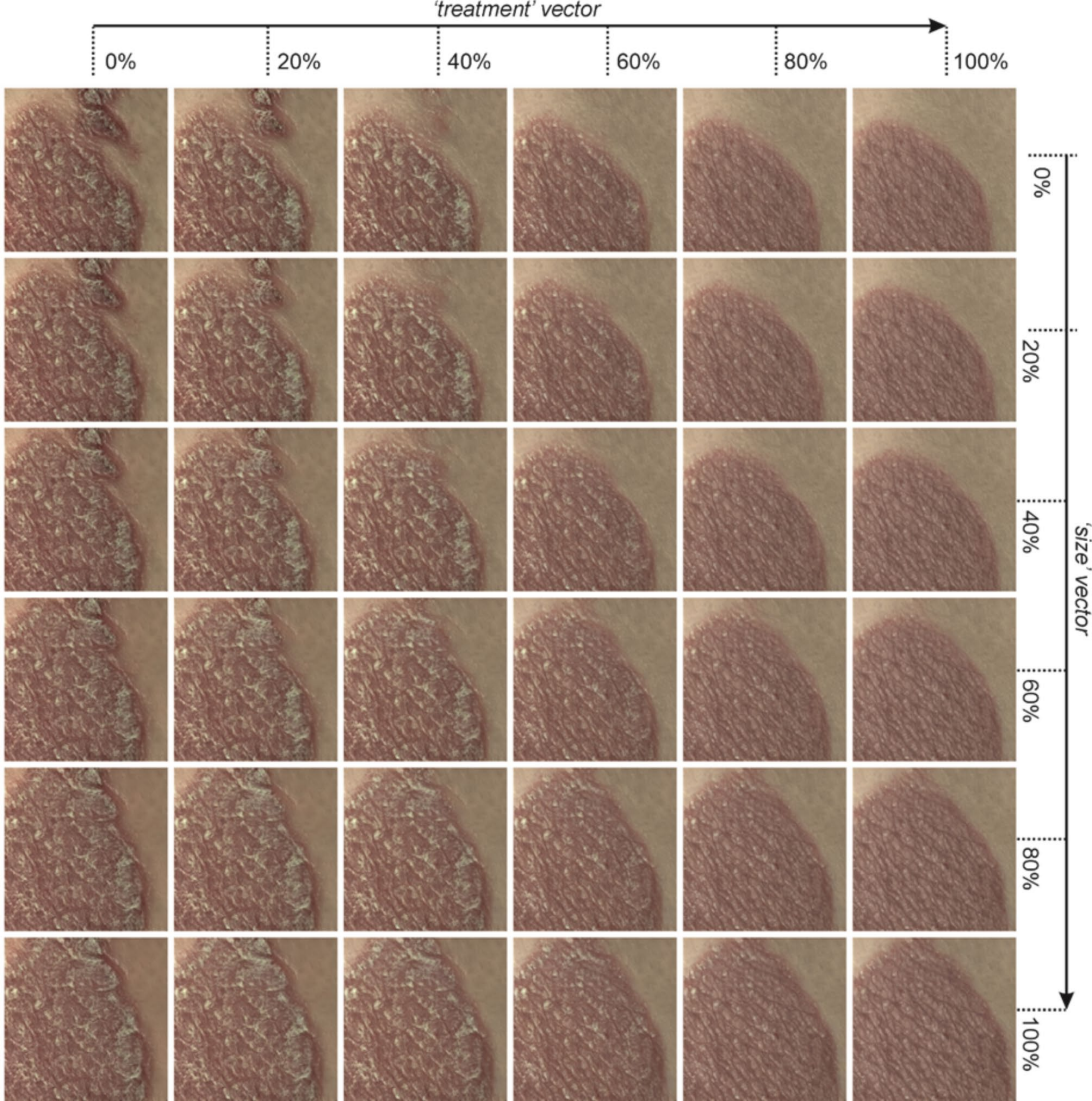
Examples of generated images with different fractions of the identified ‘treatment’ vector and a separately calculated ‘size’ vector.

Figure 7 shows the effect of adding fractions of 20%, 40%, 60%, 80% and 100% of both the ‘treatment’ vector and the ‘size’ vector to a generated image. As shown in the figure, the ‘size’ vector could be used to counteract the slight change in size of the psoriasis plaque when the ‘treatment’ vector is added. In this case, the ‘size’ vector was calculated by identifying the average w-space vector between 54 generated images of moderate and severe plaques that covered less than 50% of the image and 55 generated images of moderate and severe plaques that covered more than 50% of the image. As the size vector was derived by identifying the latent space direction that differentiated these two groups of images, it was correlated with the degree of plaque coverage on each generated image. This provides another proof-of-concept demonstration of the use of w-space vectors for manipulating specific features in a controlled and reproducible manner. Indeed, w-space vectors could be identified for any clinically useful feature, for example changes in skin appearance due to UVB damage, variations in melanin due to sun exposure, or even the amount of hair.

Whilst latent w-space vectors for severity and psoriasis plaque size were identified in this work, the anticipated clinical application of this approach would use latent w-space vectors that corresponded to specific types of treatment options and be normalised for specific time intervals. This would allow the use of GAN-generated hypothetical representations of severity changes based on latent vector manipulations to demonstrate to patients likely alterations in their psoriasis under different treatment plans and for different time periods, hence supporting clinical decision making, particularly in cases where trade-offs and side-effects (such as skin cancer risks for extended UVB treatment) might be an important consideration. Whilst, here, the images that were transformed were generated by the neural network, there are a range of methods for inserting images into a generative network^17^ and hence a photograph of a patient’s skin/skin condition could be used as the starting image. Indeed, as approximately 100 images can be generated per second using a desktop PC with a suitable graphics processing unit (GPU) using this method, the technique presented here could potentially provide real-time predictive visualisation from a live video feed from a patient with multiple psoriatic plaques (or other rashes and/or lesions).

## Conclusions

A StyleGAN neural network was trained to generate synthetic images of psoriasis and healthy skin, after being trained on 375 photographs of patients taken in a clinical setting. A latent w-space vector was identified that allowed the degree of severity of the psoriasis in the generated images to be modified. A second latent w-space vector was identified that allowed the size of the psoriasis plaque to be modified. With appropriate training data, such an approach could see a future application in a clinical setting where a patient is able to observe a prediction for the appearance of their skin disorder and/or ‘normal’ skin, under a range of treatments and after different time periods, hence allowing an informed and data-driven decision on optimal treatment to be determined. This application would therefore have relevance to both the treatment of skin diseases using pharmacological and/or physical treatment modalities and to the cosmetic industry using cosmeceuticals and/or physical modalities to improve the appearance of skin. Future work in this area will focus on embedding real-life photographs into the model so that the generated images can be compared with pre- and post-treatment clinical images in order to evaluate how the model performs compared with real treatment responses. Limitations of this study include a relatively small sample size of 375 images and therefore it is not wholly representative of the spectrum of individuals with psoriasis (age, sex, ethnicity), the subtypes of psoriasis (plaque, guttate, flexural) and the full spectrum of disease severity within psoriatic lesions. Diversifying the training data set so that the model includes individuals from different skin tone groups and images of other forms of psoriasis will be required in our future work prior to its use in clinical practice for all patients with psoriasis.

## Electronic supplementary material

Below is the link to the electronic supplementary material.

Supplementary Material 1

## Data Availability

Access to the dataset is restricted as it contains patient images. Applications to access the data, made by bona fide researchers, will be considered by the data access committee, and should be directed to e.healy@soton.ac.uk. All associated code and weights will be deposited on the University of Southampton PURE repository, as per UK open access requirements, and will be available to researchers on reasonable request. The code for the neural network can be found at: https://github.com/NVlabs/stylegan2-ada-pytorch.
