# Supplementary material for: Modifying the severity and appearance of psoriasis using deep learning to simulate anticipated improvements during treatment
Source: Sci Rep. 2025 Mar 3;15:7412. doi: 10.1038/s41598-025-91238-y (PMC11876654; doi:10.1038/s41598-025-91238-y)
Supplement: Supplementary file 1 — Supplementary Material 1 [file 41598_2025_91238_MOESM1_ESM.docx]

**Modifying the severity and appearance of psoriasis using deep learning to simulate anticipated improvements during treatment.**

**Supplementary information**

**Supplementary Figure 1.**


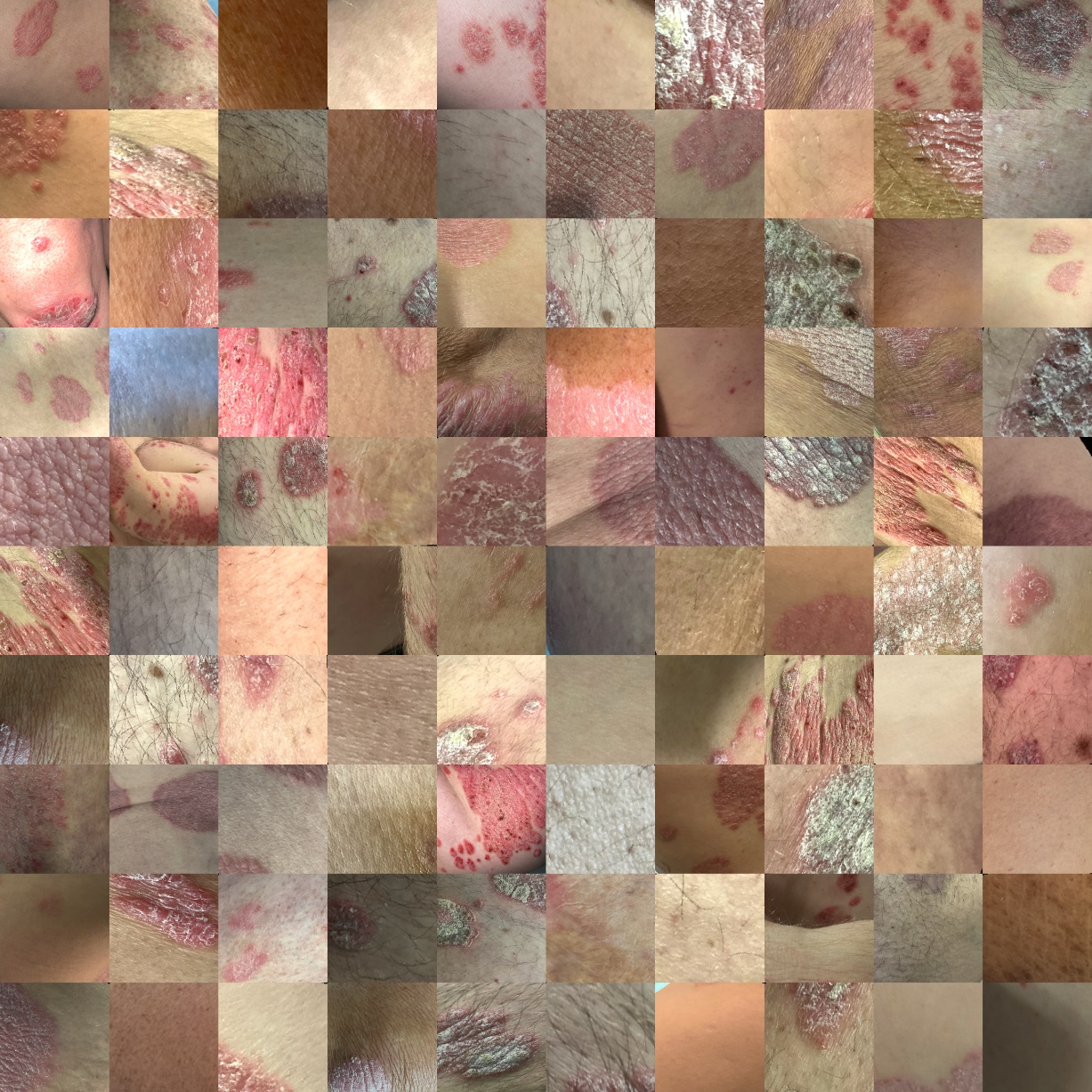


Supplementary Figure 1. 100 randomly selected examples of training images.


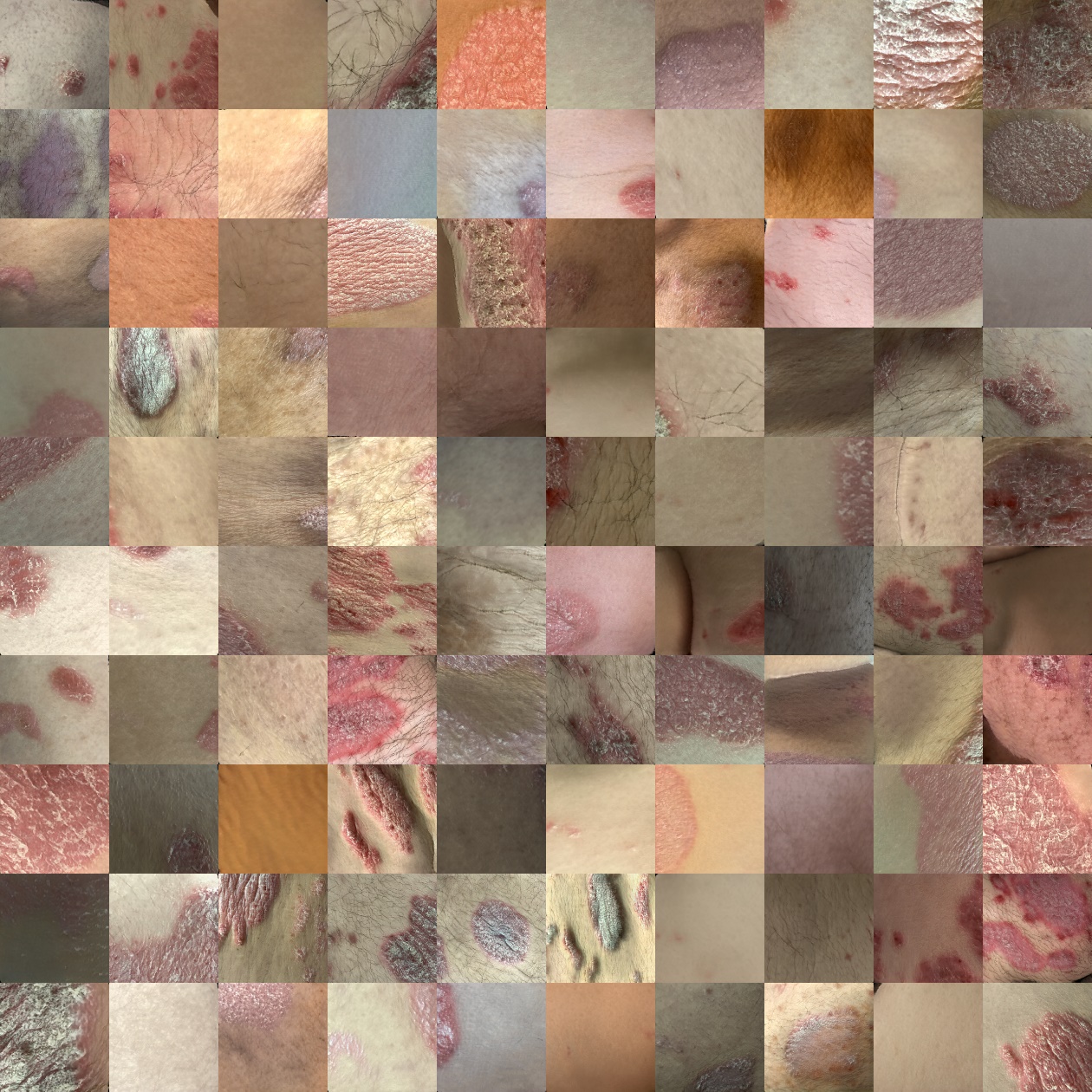
**Supplementary Figure 2.**

Supplementary Figure 2. 100 randomly selected examples of generated images.

**Supplementary Figure 3.**

**
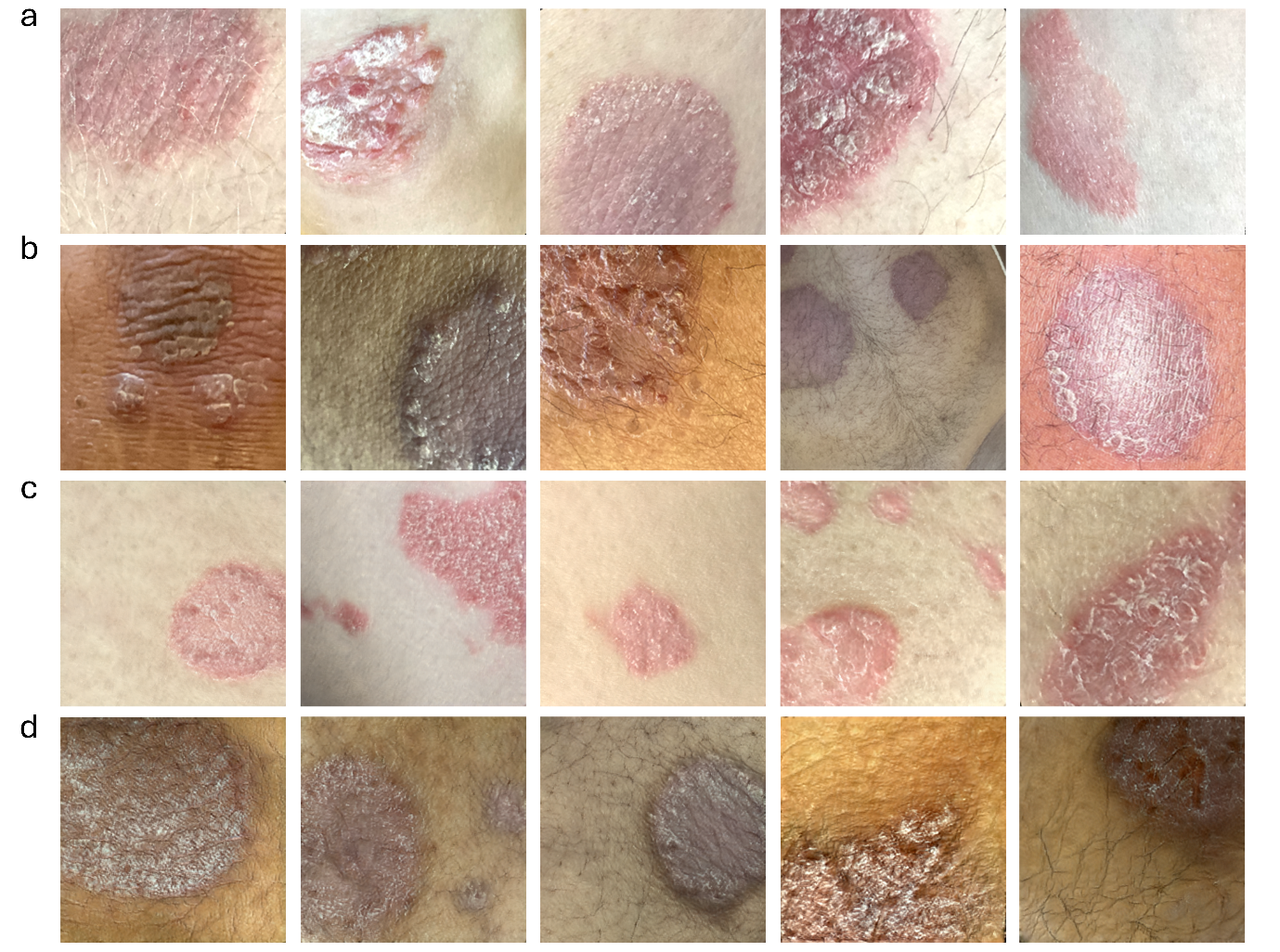
**

Supplementary Figure 3. Clinical images of psoriasis in (**a**) lighter skin and (**b**) darker skin, and StyleGAN-generated images in (**c**) lighter skin and (**d**) darker skin.
